# Effects of Lifestyle, Diet, and Body Composition on Free Testosterone and Cortisol Levels in Young Men

**DOI:** 10.3390/nu17233772

**Published:** 2025-11-30

**Authors:** Dominika Mazurkiewicz, Robert Gajda, Jagoda Ambrozik-Haba, Wiktoria Bożek, Maja Ceremuga, Paweł Serek

**Affiliations:** Department of Human Nutrition, Faculty of Biotechnology and Food Sciences, Wrocław University of Environmental and Life Sciences, Chełmońskiego 37, 51-630 Wrocław, Poland; dominika.mazurkiewicz@upwr.edu.pl (D.M.); robert.gajda@upwr.edu.pl (R.G.); jagoda.ambrozik-haba@upwr.edu.pl (J.A.-H.); 121385@student.upwr.edu.pl (W.B.); 121423@student.upwr.edu.pl (M.C.)

**Keywords:** testosterone, cortisol, lifestyle, nutrition, stress, sleep, alcohol, smoking

## Abstract

**Background/Objectives**: Testosterone and cortisol are key regulators of metabolic, psychological, and physiological responses to environmental and lifestyle factors. This study aimed to analyze the relationship between free testosterone and cortisol concentrations and dietary patterns, stress levels, sleep quality, physical activity, and body composition in healthy young men (aged 18–35 years). **Methods**: This study included 40 volunteers who met our inclusion criteria. They underwent anthropometric measurements, body composition analysis, and biochemical determination of serum free testosterone and cortisol concentrations. Additionally, participants completed a set of validated questionnaires: a questionnaire regarding the frequency of consumption of specific foods and stimulants, a 3-day food diary, the PSS-10, the Holmes and Rahe Scale, the PSQI, and the Baecke Physical Activity Questionnaire. **Results**: Free testosterone concentration in blood was negatively correlated with body fat content and positively correlated with the percentage of energy, protein, fat, sodium, and folic acid requirements. Morning blood cortisol levels negatively correlated with body weight and height. Higher intakes of cholesterol, folic acid, and vitamin A resulted in statistically significant reductions in cortisol levels. A significant correlation was identified between poor sleep quality and low cortisol levels, particularly among men aged < 26 years. A positive correlation was also found between leisure-time physical activity and testosterone levels, particularly in the older group. Furthermore, a higher body weight and greater muscle mass were correlated with lower cortisol levels. **Conclusions**: These results provide a starting point for further research on neuroendocrine mechanisms in active individuals exposed to environmental stress.

## 1. Introduction

Testosterone and cortisol are steroid hormones that play key roles in regulating reproductive, metabolic, and psychophysiological functions in men. Testosterone, synthesized mainly by Leydig cells in the testes, is responsible for the development of sexual characteristics, libido, spermatogenesis, maintenance of muscle mass, bone mineral density, and overall metabolic efficiency [1,2]. Cortisol, secreted by the adrenal cortex in response to activation of the hypothalamic–pituitary–adrenal (HPA) axis, regulates the body’s response to stress, affects glucose and protein metabolism, and has anti-inflammatory and immunosuppressive effects [3,4].

Both testosterone and cortisol synthesis are subject to circadian rhythms and are sensitive to environmental and behavioral factors [5,6]. It has also been found that excess visceral adipose tissue can disrupt the functioning of hormonal axes by increasing aromatase activity and inflammatory cytokine production, leading to decreased testosterone and/or increased cortisol concentrations [7,8].

Lifestyle factors such as physical activity, sleep quality, diet and stimulant use (e.g., alcohol, caffeine) also affect hormone balance. It has been shown that intense physical exercise can temporarily increase testosterone levels, while chronic training overload or mental stress can lead to an increase in cortisol and a secondary decrease in testosterone [9,10]. The composition of daily diet also plays an important role. Adequate supply of energy, macronutrients, vitamins, and minerals is essential for proper hormone synthesis. Deficiencies in zinc, magnesium, or vitamin D can lower testosterone levels, whereas high consumption of simple sugars or excessive calorie restrictions can lead to increased cortisol secretion [11,12].

Despite numerous studies in the literature, many relationships between lifestyle, nutritional status, body composition, and testosterone and cortisol concentrations in young male populations remain unclear or insufficiently studied. To date, most studies have focused on clinical populations or middle-aged or older individuals. There is a need for an in-depth analysis of hormonal relationships among young adults who are developing lasting health and metabolic patterns.

This study aimed to investigate the relationship between selected lifestyle factors (physical activity, sleep quality, stress, diet, and stimulants) and body composition and testosterone and cortisol concentrations in a population of young, healthy males.

## 2. Materials and Methods

### 2.1. Study Group

Forty healthy men aged 19–32 years from Poland participated in the study. Recruitment was non-random and based on voluntary participation. The inclusion criteria were as follows: male sex; age 18–35 years; no diagnosed metabolic or autoimmune diseases or infertility; and no use of drugs that could affect testosterone or cortisol levels. The participants were informed about the study and provided written informed consent to participate.

### 2.2. Blood Sample Collection and Analysis

Participants were referred to the Diagnostyka S.A. laboratory network for blood serum collection and biochemical analysis. This medical laboratory is accredited by the Polish Centre for Accreditation (PCA), accreditation number AM 003, confirming compliance with rigorous quality and competence standards. Blood samples were collected in the morning (between 7:00 and 9:00 a.m.) after an overnight fast, with participants instructed to avoid intense physical activity and stress on the day prior to testing. Free testosterone concentration was determined by chemiluminescent immunoassay (CLIA) using the MAGLUMI Free Testosterone kit (Snibe Diagnostic, Shenzhen, China) on the Maglumi X8 automated analyzer, with results expressed in picograms per milliliter (pg/mL). Serum cortisol concentration was measured by electrochemiluminescence immunoassay (ECLIA) using the Cobas e analyzer (Roche Diagnostics, Mannheim, Germany) and the corresponding reagent kit, according to the manufacturer’s instructions, with results expressed in micrograms per deciliter (µg/dL).

Both assays are validated by the manufacturers against reference methods to ensure analytical accuracy and reliability. Direct measurement of free testosterone was employed rather than calculated from total testosterone and sex hormone-binding globulin (SHBG) to directly reflect the physiologically active hormone fraction available to target tissues.

### 2.3. Anthropometric and Body Composition Measurements

Body height was measured using a Charder MS 4971 height gauge (Charder Medical, Taichung, Taiwan), and body weight and composition were measured using a certified InBody 270 analyzer (InBody Co., Ltd., Seoul, Republic of Korea), which uses the bioelectrical impedance method [13,14]. Waist circumference was also measured. Before the measurements, the participants were instructed to fast or refrain from eating for at least 2–3 h. The analyzer provided data on, among other things, body weight, fat mass, muscle mass, percentage of fat mass and segmental body analysis.

### 2.4. Survey Methods

A structured questionnaire based on the KomPAN tool (Committee of Human Nutrition, Polish Academy of Sciences, Poland) supplemented with original questions was used to collect data on lifestyle and eating habits. The questionnaire allowed for the assessment of the frequency of consumption of various food groups and calculation of the pHDI-10 (healthy diet index) and nHDI-14 (unhealthy diet index) indicators. The questionnaire also included questions on the use of stimulants.

The level of physical activity was assessed both on the basis of the Physical Activity Level (PAL) self-classification and using the validated Baecke Physical Activity Questionnaire, the results of which allow the calculation of the index of professional, sports, and leisure activities [15].

The Holmes and Rahe Stress Rating Scale (SRRS) was used to assess stress levels. This tool measures stress based on the number of stressful life events over the past year. The scale contained 43 life events, which were assigned numerical values corresponding to the stress caused by a given event. After completing the questionnaire, the points awarded for the selected events were added and interpreted in a way that allowed the risk of illness over the next two years to be assessed as a percentage [16]. The PSS-10 (Perceived Stress Scale) questionnaire was used to assess the intensity of stress related to one’s life situation over the last month. The scale consists of ten questions about subjective perceptions of stress, everyday problems, and ways of coping with them. The higher the score, the higher the stress intensity in the previous month.

Sleep quality was assessed using the standardized PSQI (Pittsburgh Sleep Quality Index) questionnaire. The interpretation of the results grouped the respondents into three groups in terms of sleep quality: good, reduced, or serious sleep problems [17].

### 2.5. Dietary Notation Method

In addition, the participants kept a 3-day food diary covering two working days and one day off. The recorded data were entered into the Kcalmar.pro diet software (Hermax sp. z o.o., Lublin, Poland; https://kcalmar.com/dietetyk/, accessed on April–June 2025). Based on these data, the intakes of energy, macronutrients, vitamins, and minerals were estimated. The calculations took into account the target body weight (based on the Lorentz formula), basal metabolic rate (BMR, according to the Harris-Benedict formula), and PAL coefficient, which allows the total metabolic rate (TMR) to be determined. The intake was analyzed in relation to the nutritional standards of the Polish population [18]. Recommendations regarding the intake of saturated fats, simple sugars, and the ratio of omega-6 to omega-3 fatty acids were also assessed.

### 2.6. Statistical Analysis

The collected data were coded and entered into StatSoft’s Statistica 13.3 software. The normality of the distribution of variables was checked using the Shapiro–Wilk test. Depending on the distribution, parametric (Pearson’s correlation, ANOVA) or non-parametric (Spearman’s correlation, Kruskal–WallisKruskal-Wallis test, and median test) tests were used. Statistical significance was set at *p* < 0.05. The strength of the relationship was determined based on the applicable correlation criteria.

## 3. Results

### 3.1. Hormones and Diet

#### 3.1.1. Testosterone

This study analyzed the effects of diet on testosterone levels in male subjects (Table 1). In both the entire group (aged 19–32) and the younger group (aged ≤ 25 years), free testosterone levels correlated significantly with dietary energy and fat intake. In the older group (≥26 years), higher fulfillment of the dietary protein requirement was associated with higher hormone concentrations. None of the dietary indices showed a statistically significant relationship with free testosterone concentration. In the case of the healthy diet index, the correlation coefficients were positive, which suggests a probable direction of change. The effect of an unhealthy diet index on free testosterone concentration appears ambiguous.

This study also examined the effect of the consumption of selected minerals on testosterone levels among male subjects (Table 2). Free testosterone levels in the blood were significantly correlated only with the fulfillment of dietary sodium requirements. No statistically significant correlations were observed for the other minerals, but the direction of change was evident, with each of the correlation coefficients taking positive values, which suggests that a higher supply of the minerals analyzed may be associated with higher free testosterone concentrations in the blood.

Table 3 shows the relationship between dietary vitamin intake and free testosterone concentration in the blood among the men studied. In the older group, a clear, statistically significant positive correlation was observed between the percentage of recommended daily allowance of folic acid and testosterone concentrations. The fulfillment of the recommended daily allowance for other vitamins did not show any statistically significant correlations or clear trends in relation to testosterone.

#### 3.1.2. Cortisol

This study assessed the relationship between diet and cortisol concentration in male participants (Table 4). Healthy and unhealthy diet indices and the intake of individual dietary macronutrients did not show a significant relationship with the hormones studied except for cholesterol younger men. It is worth noting that the correlation coefficient for cholesterol showed the opposite trend in the older group (≥26 years). A higher intake of both total fat and individual fatty acids was associated with a decrease in cortisol concentration, although this relationship was not statistically significant.

Table 5 shows the relationship between dietary mineral intake and blood cortisol levels among male subjects. None of the correlation coefficients was statistically significant. However, it can be noted that each of the coefficients took negative values, which would suggest that higher mineral intake may have contributed to lower blood cortisol concentrations.

Table 6 shows the relationship between dietary vitamin intake and blood cortisol levels in male participants. One of the most important statistically significant observations was the relationship between blood cortisol levels and folic acid and vitamin A intake among the male participants. Higher vitamin A intake was significantly associated with a decrease in hormone concentration in the entire group. This correlation was even stronger in the younger participants and showed the strongest correlation among all the tests performed. A similar relationship was observed for folic acid. A higher intake of this vitamin among men under 25 years of age was associated with a decrease in serum cortisol concentration. The supply of other fat-soluble vitamins (vitamins D and E) did not correlate statistically significantly with the concentration of the hormone in question but showed a negative direction of change.

### 3.2. Hormones and Stimulants

#### 3.2.1. Testosterone

**Figure 1:**
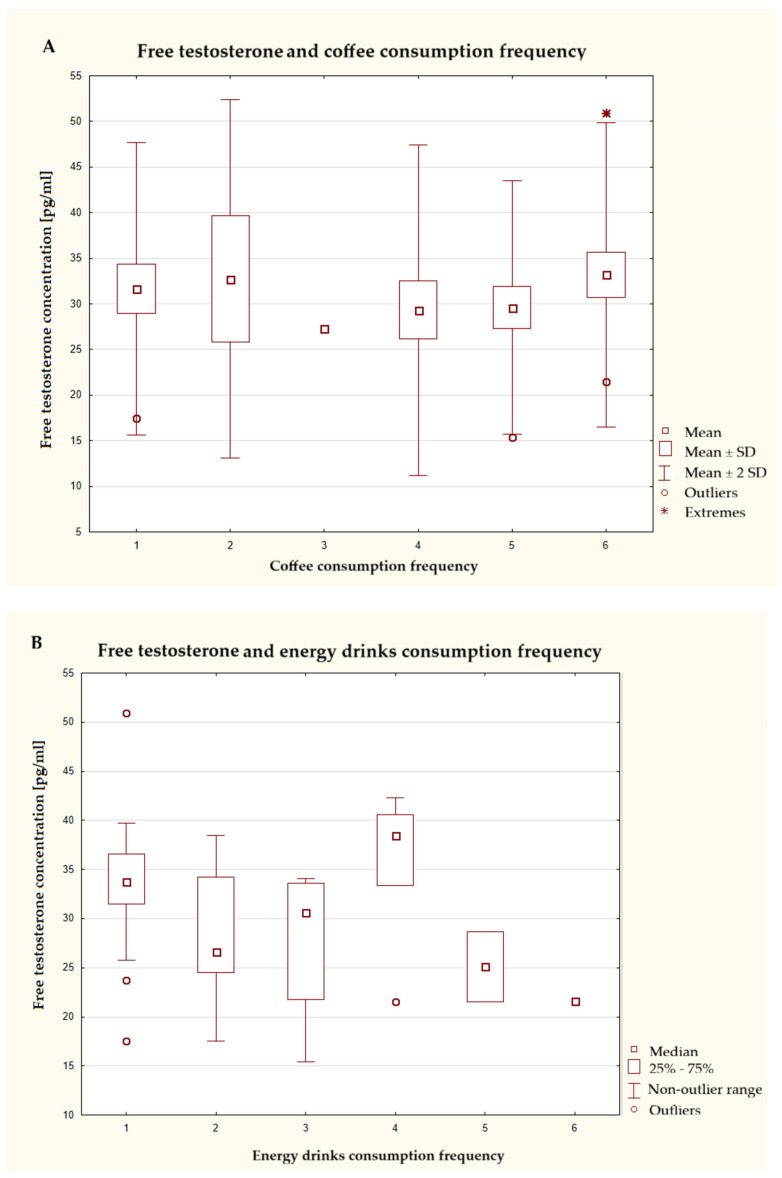

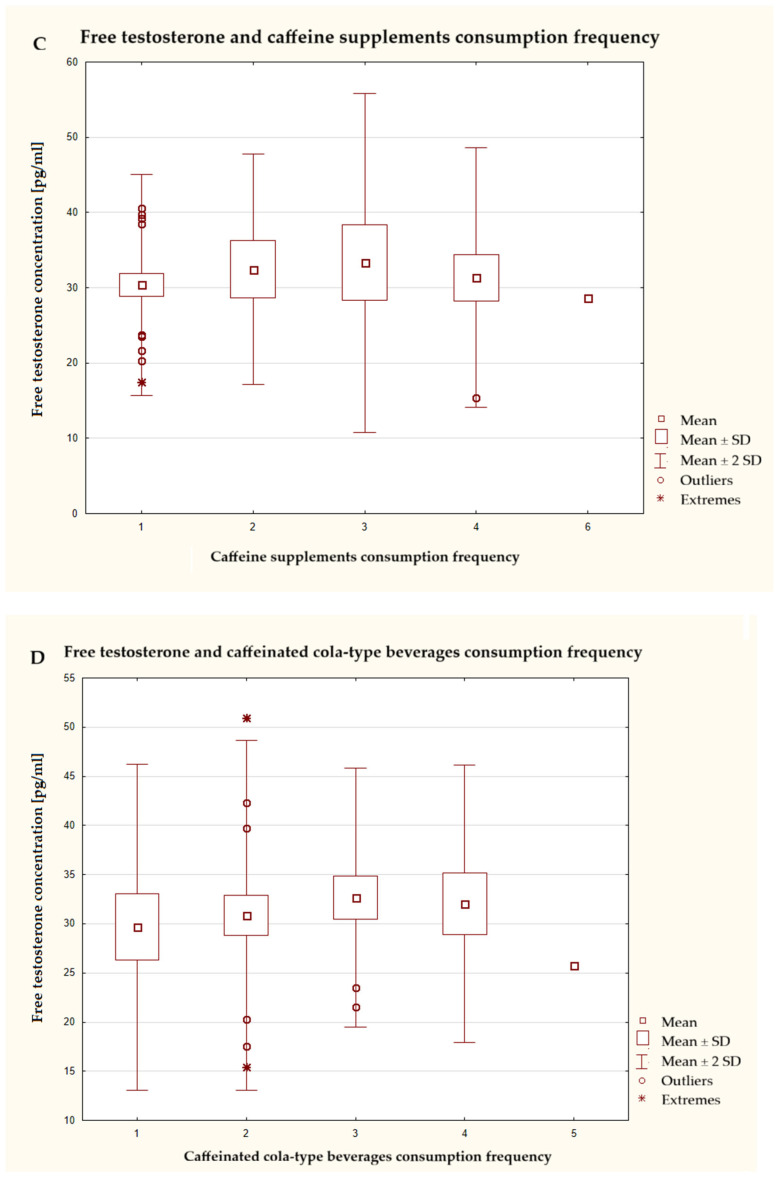
(**A**–**D**) Characteristics of free testosterone concentration depending on the frequency of consumption of caffeinated products (*n* = 40). The responses presented in the graphs using numbers should be read according to the following legend: 1 = never, 2 = 1–3 times a month, 3 = once a week, 4 = several times a week, 5 = once a day, and 6 = several times a day. Coffee—ANOVA test at a significance level of α = 0.05:1.0889), *p* = 0.3842; energy drinks—Kruskal–Wallis test at a significance level of α = 0.05:10.2213, *p* = 0.0692; dietary supplements—ANOVA test at a significance level of α = 0.05:0.1876, *p* = 0.9433; cola—ANOVA test at a significance level of α = 0.05:0.2574, *p* = 0.9031.

This study assessed the effects of caffeine consumption from various sources on free testosterone levels in male subjects (Figure 1). No clear upward or downward trend was observed depending on the frequency of coffee consumption. Slightly higher average concentrations of this hormone were observed in the group consuming coffee 1–3 times a month and several times a day. The widespread of data indicates a high individual variability. In the case of energy drinks, differences in average testosterone levels were observed between the groups, with the highest median recorded among those who reported consuming these drinks several times a week. In contrast, the lowest average values were observed in the groups with the highest frequency of consumption, once a day and several times a day, suggesting a downward trend in testosterone levels with increasing consumption of this type of drink. In the case of dietary supplements containing caffeine, no statistically significant differences were observed between the groups, and there was a significant spread of data and numerous outliers, especially in the group that had never consumed these foods. In the case of consumption of caffeinated cola drinks, low group variability and, at the same time, significant individual variability can also be observed. Moreover, the range of the standard deviation indicates a very wide range of data. In the group consuming these types of beverages 1–3 times a week, most outliers and extreme values were observed.

**Figure 2:**
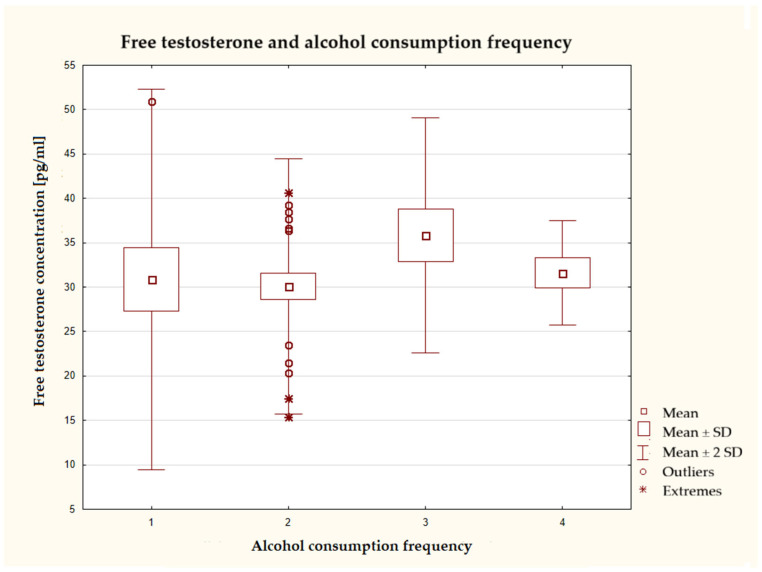
Characteristics of free testosterone concentration depending on the frequency of alcohol consumption (*n* = 40), ANOVA test at a significance level of α = 0.05:0.7319, *p* = 0.5398; the answers presented in the graphs below using numbers should be read according to the following legend: 1 = never; 2 = 1–3 times a month; 3 = once a week; 4 = several times a week; 5 = once a day; and 6 = several times a day.

The highest average free testosterone concentration was found in the group that consumed alcoholic beverages once a week, but no statistically significant differences were observed between the groups (Figure 2). The group that consumed alcohol most frequently (several times per week) was characterized by the smallest data dispersion.

**Figure 3:**
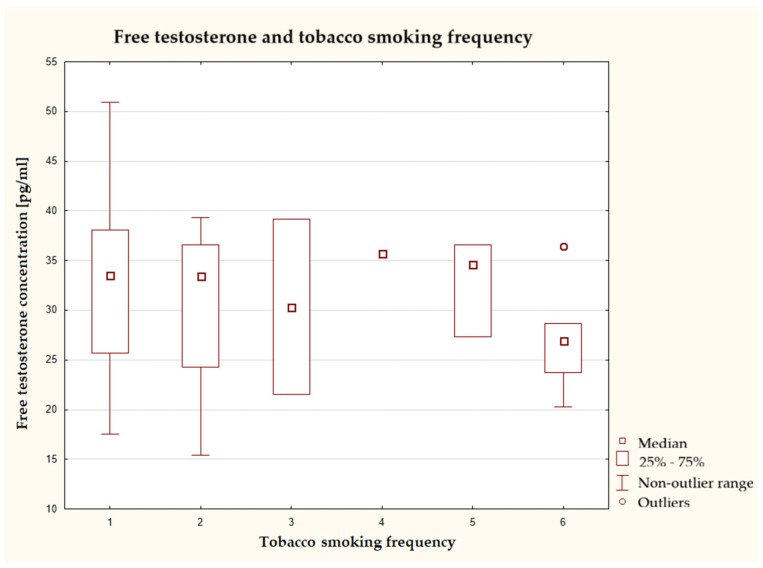
Characteristics of free testosterone concentration depending on tobacco smoking frequency (*n* = 40), Kruskal–Wallis test at a significance level of α = 0.05: 2.6392, *p* = 0.7554. The responses presented in the graphs using numbers should be read according to the following legend: 1 = never, 2 = 1–3 times a month, 3 = once a week, 4 = several times a week, 5 = once a day, and 6 = several times a day.

The lowest average concentration of free testosterone was found in the group that smoked at least several times a day. Moreover, this group was characterized by a narrow range between the first and third quartiles (Figure 3). In contrast, the widest concentration range was found in the non-smoking group. Although the test did not show that these results were below the significance threshold, it could suggest a certain trend.

**Figure 4:**
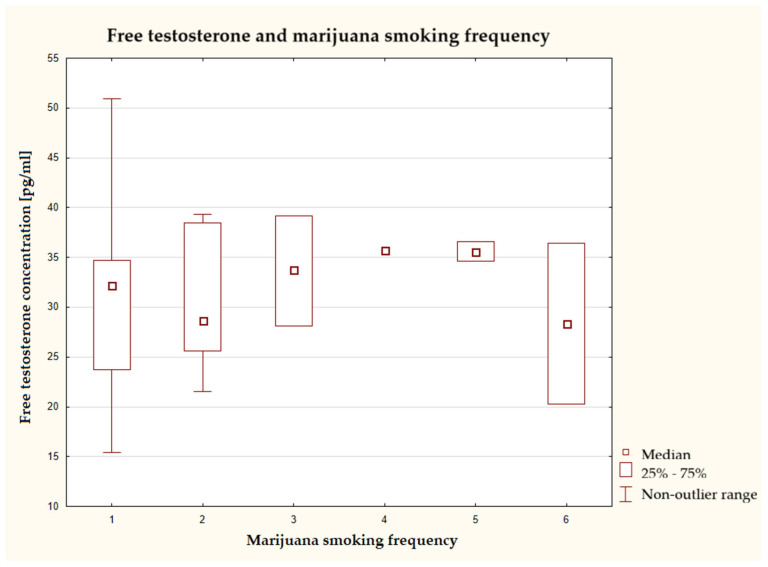
Characteristics of free testosterone concentration depending on frequency of marijuana use (*n* = 40), Kruskal–Wallis test at a significance level of α = 0.05: 2.3917, *p* = 0.7927. The answers presented in the graphs using numbers should be read according to the following legend: 1 = never, 2 = 1–3 times a month, 3 = once a week, 4 = several times a week, 5 = once a day, and 6 = several times a day.

When marijuana was used several times a week or once a day, the median testosterone concentration was the highest, while frequency of use 1–3 times a month and once a day was associated with the lowest median (Figure 4). There were no statistically significant differences in variability between the groups.

#### 3.2.2. Cortisol

**Figure 5:**
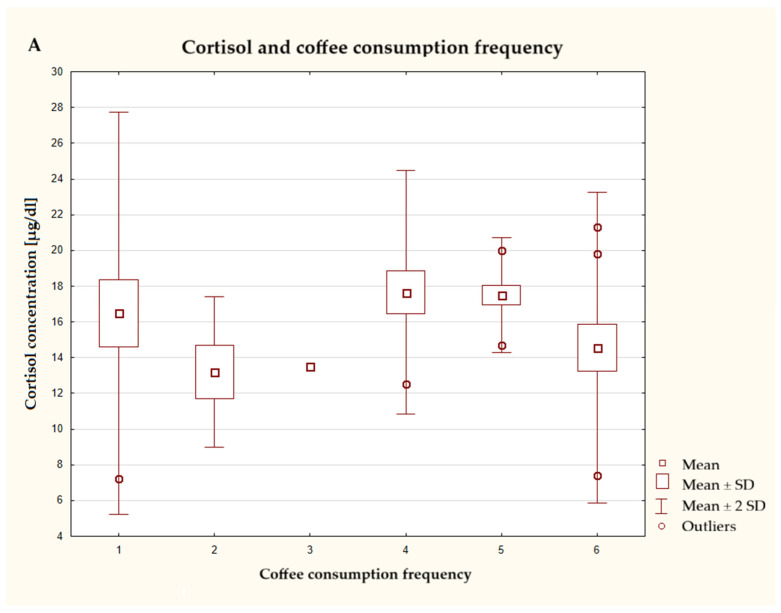

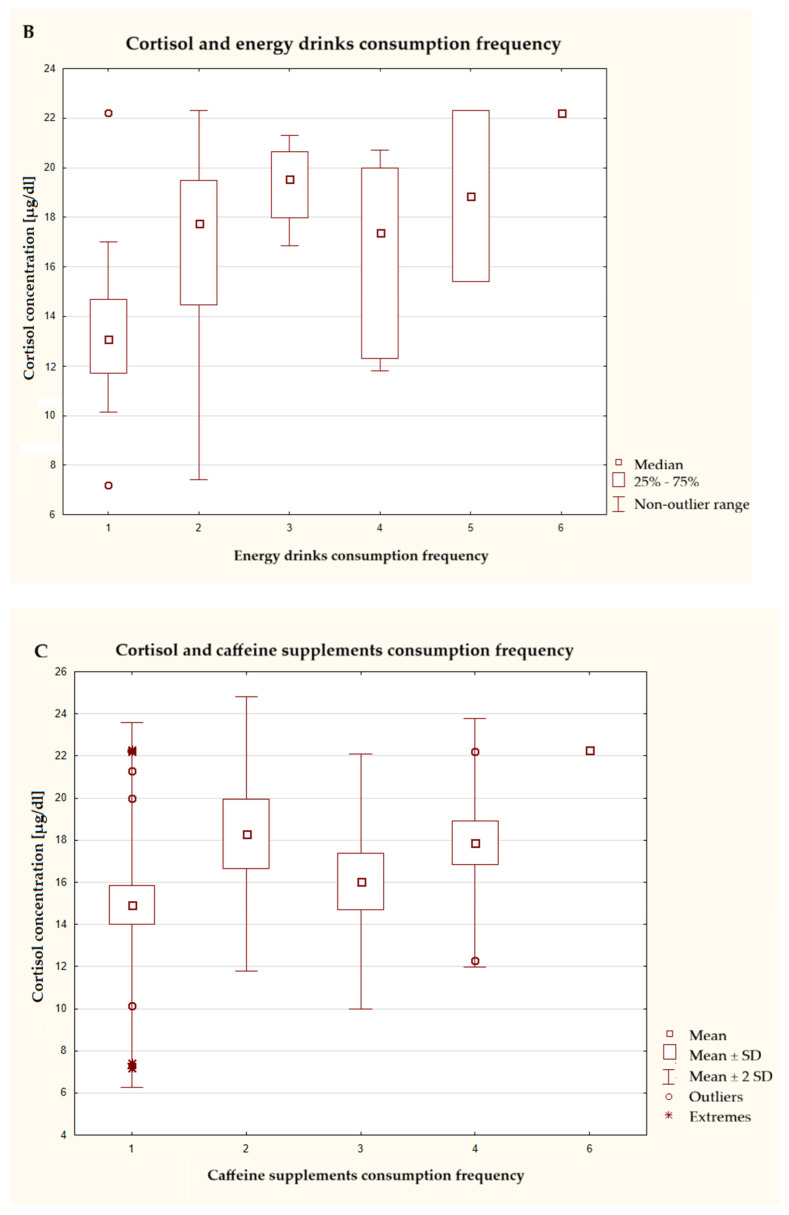

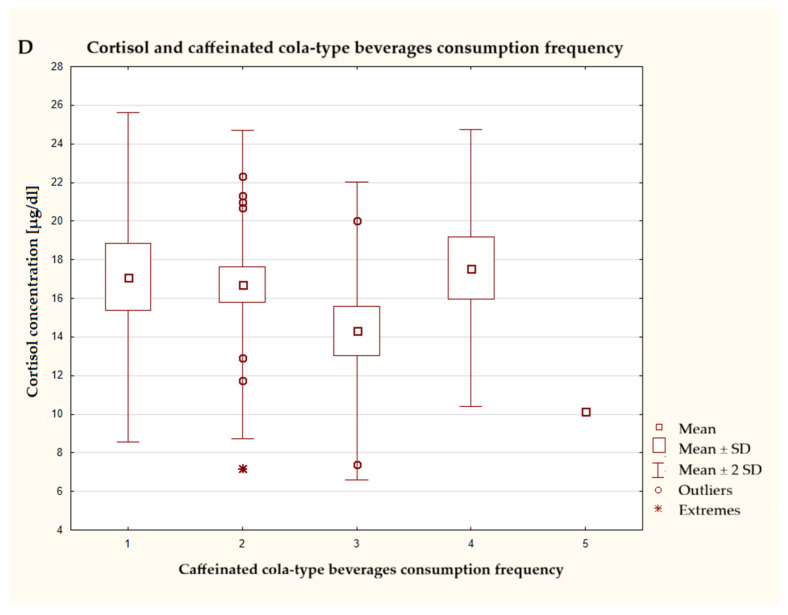
(**A**–**D**) Characteristics of cortisol concentration depending on the frequency of consumption of caffeinated products (*n* = 40), the responses presented in the graphs using numbers should be read according to the following legend: 1 = never, 2 = 1–3 times a month, 3 = once a week, 4 = several times a week, 5 = once a day, and 6 = several times a day. coffee—ANOVA test at a significance level of α = 0.05:1.0889, *p* = 0.3842; energy drinks—Kruskal–Wallis test at a significance level of α = 0.05:12.805, *p* = 0.0253; cola—ANOVA test at a significance level of α = 0.05:1.3933, *p* = 0.2564; dietary supplements—ANOVA test at a significance level of α = 0.05:1.9006, *p* = 0.1322.

This study assessed the impact of various caffeine sources on cortisol levels in male subjects. It was found that the average cortisol concentration was highest in the group that consumed coffee several times a week, once a day, and lowest in the group that drank coffee 1–3 times a month or once a week. The greatest dispersion of data, as well as numerous outliers, was observed in the two extreme groups: those who never drank coffee and those who drank it several times a day. In the case of energy drink consumption, it can be seen that the median cortisol concentration increased with the frequency of consumption of these foods. The lowest concentrations of this hormone were associated with rarest or complete non-consumption of this type of drink. The frequency of consumption of caffeinated cola did not affect the concentration of the hormones in question. Figure 5C also shows the relationship between cortisol concentration and frequency of use of dietary supplements containing caffeine. Considering the average values of this hormone concentration in individual groups, it was observed that a higher frequency of use of this supplement was associated with higher cortisol concentrations in the blood of subjects. The greatest deviations, with numerous outliers and extreme values, were observed in the group that had never consumed this type of food.

**Figure 6:**
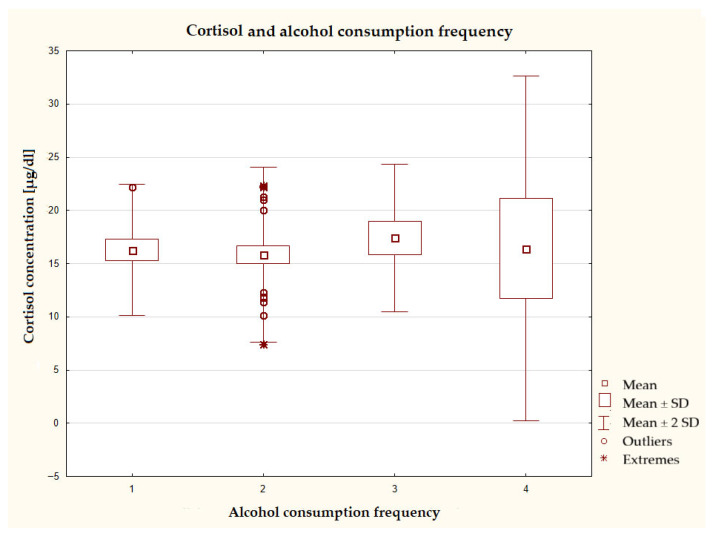
Characteristics of cortisol concentration depending on the frequency of alcohol consumption (*n* = 40), ANOVA test at a significance level of α = 0.05:0.2039, *p* = 0.8930; responses presented in the graphs below using numbers should be read according to the following legend: 1 = never, 2 = 1–3 times a month, 3 = once a week, 4 = several times a week, 5 = once a day, and 6 = several times a day.

The graph comparing cortisol concentration with the frequency of alcohol consumption shows that in the group drinking alcohol most often, that is, several times a week, the spread of cortisol concentration values was the highest among all groups (Figure 6). The smallest deviations from the mean were observed in the group that did not consume alcoholic beverages. This group, together with the group consuming these beverages 1–3 times a week, also showed the lowest mean concentrations of the hormones tested, although the differences in this respect were insignificant in all groups.

**Figure 7:**
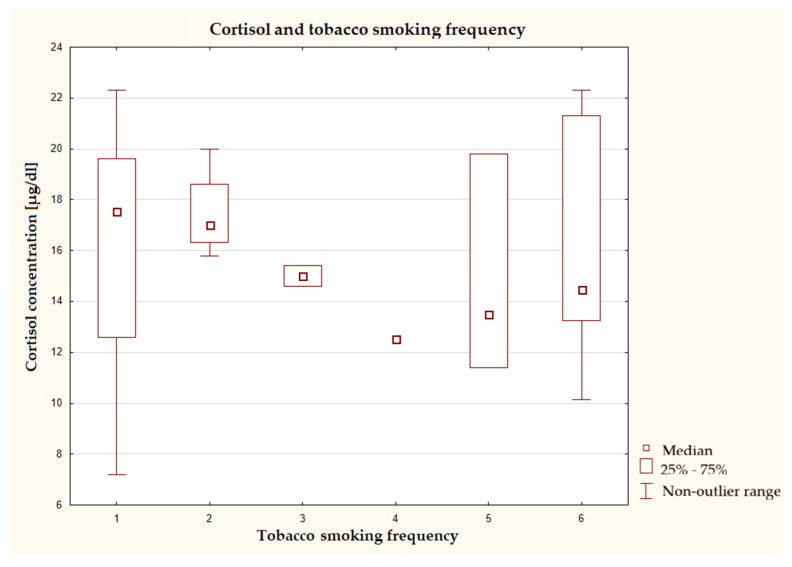
Characteristics of cortisol concentration depending on tobacco smoking frequency (*n* = 40), Kruskal–Wallis test at a significance level of α = 0.05:2.3425), *p* = 0.8000. The responses presented in the graphs using numbers should be read according to the following legend: 1 = never, 2 = 1–3 times a month, 3 = once a week, 4 = several times a week, 5 = once a day, and 6 = several times a day.

No clear trend was found between the frequency of smoking and cortisol concentration among respondents (Figure 7). The median cortisol level was relatively high in the never smokers and those who smoked 1–3 times a week and slightly lower in the groups who smoked more frequently. The range of concentrations in these groups may suggest high variability in the results.

**Figure 8:**
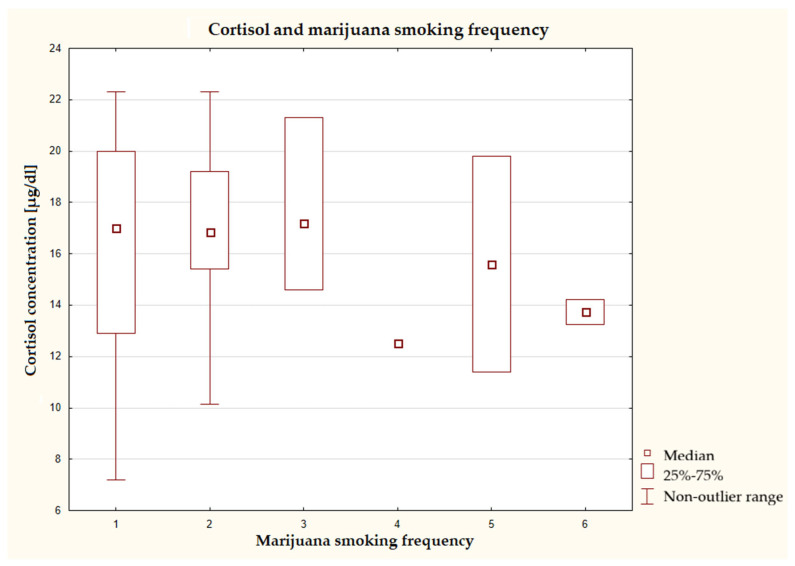
Characteristics of cortisol concentration depending on the frequency of marijuana use (*n* = 40), Kruskal–Wallis test at a significance level of α = 0.05:2.687, *p* = 0.7481. The answers presented in the graphs using numbers should be read according to the following legend: 1 = never, 2 = 1–3 times a month, 3 = once a week, 4 = several times a week, 5 = once a day, and 6 several times a day.

The analysis showed that the highest median cortisol levels were observed in individuals who had never used cannabis and in those who had used it 1–3 times a month or once a week (Figure 8). As the frequency of cannabis use increased, a downward trend in median cortisol concentration was observed. This was particularly evident in the group that used these products several times daily. In the groups using cannabis less frequently, particularly, high variability in results was observed.

### 3.3. Hormones and Body Composition

This study assessed the relationship between the body composition of male subjects and the concentrations of free testosterone and cortisol in the blood. Both body weight and height in the entire group and in the group up to 25 years of age were negatively correlated with cortisol concentration (Table 7). No statistically significant correlations were observed between these variables in the group aged > 26 years. Parameters such as BMI, waist circumference, fat, and muscle tissue did not show any clear trends.

It was also demonstrated that the only statistically significant correlation was between testosterone concentration and body fat percentage among participants over 26 years of age (Table 8). In the older group, a higher body fat content was associated with a decrease in hormone concentration. A similar trend in the older group was observed for BMI, but this relationship was not statistically significant in the study group.

### 3.4. Hormones and Stress

Statistical analysis of Spearman’s correlation between testosterone and cortisol concentrations and the results of the Holmes and Rahe Scale (stress resulting from events in the last year) and the PSS-10 scale (level of perceived stress in the last month) did not show any statistically significant relationships (Table 9). All correlation coefficients obtained ranged from −0.15 to −0.04, which indicates very weak negative correlations.

### 3.5. Hormones and Sleep

Participants’ results, assessed using the PSQI scale, ranged from good to generally poor sleep quality. None of the participants had serious sleep problems. No significant relationship was found between testosterone concentration and sleep quality, and the correlation coefficient was very weak and positive (r = 0.09). However, cortisol concentration and PSQI scores were weakly negatively correlated (r = −0.38), indicating that poorer sleep quality (higher PSQI score) may be associated with lower cortisol concentration. After analyzing the data by age group, no correlation was found in the group over 26 years of age. However, in the group of men under 26 years of age, a moderate negative correlation was found between cortisol concentration and PSQI score (r = −0.45), which may indicate a stronger relationship between poorer sleep quality and lower cortisol levels in this group. The results are summarized in Table 10.

### 3.6. Hormones and Physical Activity

No statistically significant correlations were found between cortisol concentration and any of the activity indices according to the Baecke Physical Activity Questionnaire. The correlation coefficients ranged from −0.17 to 0.04. However, a very weak negative correlation (r = −0.17) was observed between cortisol concentration and the leisure activity index, suggesting that people who are more active during their free time may have lower cortisol concentrations. In the case of testosterone, a positive correlation with physical activity during leisure time was observed (r = 0.41), indicating that people who were more active in this area had higher testosterone levels. However, no significant correlations were found between testosterone levels and professional or sporting activities. After dividing the study group by age, two significant correlations were observed among men over 26 years of age: testosterone was positively correlated with leisure time activity (r = 0.49) and professional activity (r = 0.50), that is, both correlations were moderate. No significant correlations were found in the younger group (≤26 years of age). The results are summarized in Table 11.

## 4. Discussion

The main objective of this study was to examine the relationship between body composition, diet, physical activity, stress, sleep quality, testosterone, and cortisol levels in young men. To date, most studies have focused on the analysis of a single variable and its effect on selected hormones. Analysis of the collected data revealed several interesting relationships; however, some of the results did not reach statistical significance, which may be due to sample size limitations or weak correlations.

### 4.1. Testosterone

In our study, total protein intake was positively correlated with free testosterone levels in men aged 26–33. Only few studies have analyzed this relationship in healthy young men. To date, protein intake has been studied mainly in the context of obesity or sports nutrition [19,20,21]. One study evaluated whether a high-protein reduction diet would be more effective than a high-carbohydrate diet in terms of, among other things, improving free testosterone levels. However, it was found that weight loss in obese individuals resulted in an increase in testosterone levels regardless of the diet analyzed, which suggests that more important indicators related to this hormone may be adipose tissue or BMI [19]. However, it was not possible to recruit a sufficiently large number of obese participants for our study, and as a result, it was not possible to form two groups (with normal and abnormal body weights) whose hormone concentrations could be compared. This is probably why body weight, waist circumference, and BMI showed no correlation with free testosterone concentration [22]. Our study showed that body fat percentage was negatively correlated with testosterone levels in men aged 26–33. Similar results were obtained in the NHANES III study, which involved 1265 American men aged > 20 years. However, the study group was characterized by a significantly older age of participants and a much greater diversity of ethnic origin. As in our study, the free testosterone concentration decreased with an increase in the percentage of body fat. The study also observed a decrease in this hormone with an increase in waist circumference and BMI, which was not observed in our study [23].

The study showed a positive correlation between the percentage of energy and fat requirements met and free testosterone concentration. Most participants had energy deficiencies. This positive correlation may, therefore, be related to significant deficiencies in the group consuming too few kilocalories compared to the group that met nearly 100% of their requirements, thus providing the body with more nutrients. To the best of our knowledge, there are no studies in the literature that can be directly compared. A study by Vidić et al. assessed the effect of dietary fat intake on testosterone levels in middle-aged men who regularly practiced strength sports. One group was fed a ketogenic diet (fat intake at 75% of energy intake), whereas the other group was fed a high-fat diet (fat intake at 65% of energy intake) with a slightly higher carbohydrate intake. Protein intake was the same in both groups. A statistically significant increase in the free testosterone concentration in the blood was observed in both groups. This could potentially indicate a beneficial effect of fat on blood testosterone levels [24]. A study by Fantus et al. also assessed the effect of fat intake on testosterone levels in men. A low-fat diet caused a decrease in total testosterone among the men studied [25].

Our study showed that free testosterone concentration was positively correlated with sodium intake. No scientific articles have addressed this relationship. Furthermore, this premise has not been confirmed in studies conducted using animal models. Mice fed a high-salt diet (4% NaCl) for six weeks showed reduced total testosterone levels and decreased expression of enzymes responsible for testosterone synthesis, thus obtaining the opposite result. The amount of sodium used in this experiment would be impossible to obtain in the human diet; therefore, these reports should be interpreted with caution [26].

Our study showed a statistically significant positive relationship between dietary folic acid intake and free testosterone concentration in the blood of men aged > 26 years. No articles in the scientific literature discuss this relationship. The relationship between folic acid and testosterone was studied in the context of supplementation of this vitamin on the total testosterone concentration in the blood. One intervention study evaluated the effect of folic acid and zinc supplementation on semen parameters and, among other things, total testosterone in fertile and infertile men. The study observed a statistically significant increase in sperm concentration, but the intervention had no effect on testosterone concentration [27]. A meta-analysis evaluating the effects of folic acid supplementation or a combination of folic acid and zinc on semen parameters and hormone concentrations showed that folic acid and zinc supplementation had no statistically significant effect on total testosterone concentration [28]. Similarly to the above-mentioned studies, our study did not show a statistically significant effect between dietary zinc intake and free testosterone concentration.

Our study did not show a significant relationship between the frequency of caffeine, alcohol, tobacco, and marijuana use and the concentration of free testosterone in the blood serum. A meta-analysis from 2024 showed that chronic alcohol consumption significantly reduces the concentration of free testosterone in the blood of healthy men. This effect was not observed in patients with alcoholism [29]. The authors did not clearly define chronic alcohol consumption, which makes it difficult to directly compare their results with those of our study. The lack of significant correlations in our study may be due to the fact that only a small proportion of the study group reported frequent consumption of alcoholic beverages. Similar results to those of our study were found in a study on smoking conducted on European men aged 40–79. Although total testosterone was significantly elevated among smokers, free testosterone concentrations did not change significantly [30]. The data obtained on marijuana use did not correspond to scientific reports. A cohort study of 1215 healthy men of reproductive age showed that free testosterone and total serum testosterone concentrations were 7% higher in men who used marijuana [31]. Other studies in this area focused on total testosterone, but showed a similar trend. A study of 5146 men found that those who had ever used THC had higher serum testosterone concentrations, with the largest increase observed in men who used THC 2–3 times a month [25]. Similar results were obtained in another study, which found that subjects who smoked marijuana more frequently had 8% higher serum testosterone concentrations; however, the authors pointed to a potential link between higher testosterone levels and a greater propensity for risky behavior, such as drug use [32].

In men over 26 years of age, this correlation was stronger, and a positive correlation with professional activity was demonstrated. The results suggest that higher physical activity, both at work and during leisure time, is associated with higher testosterone levels, which is confirmed by previous studies showing that physical exercise stimulates testosterone secretion. Meta-analyses show that testosterone levels increase immediately after moderate and intense exercise, but not after light exercise [33]. Similarly, Hayes et al. showed that appropriately selected sports activities promote an increase in both total and free testosterone levels [34].

Our study found no association between testosterone concentration and subjectively perceived stress or stress assessed based on objective life events. Ilkevič’s results indicate that higher levels of free testosterone are associated with lower stress perception only in individuals with low cortisol levels, which is consistent with the dual-hormonal hypothesis that the relationship between testosterone and cortisol determines the effects of psychosocial stress [35]. A study involving 718 men treated for infertility showed that higher levels of subjective stress reduced sperm count and quality but were not significantly associated with sex hormone concentrations (including testosterone). Stress negatively affects semen parameters, mainly through oxidative and inflammatory mechanisms rather than hormonal mechanisms [36]. In Marceau’s study on adolescents, an increase in pandemic stress was associated with a parallel increase in cortisol, testosterone, and DHEA concentrations, especially in boys with higher exposure to stress [37]. The links between stress and testosterone are complex and depend on the type of stress, population, and biological mechanisms, as confirmed by the differing results of various studies.

Our study did not find a relationship between testosterone levels and PSQI scores. No studies in the literature have directly analyzed the correlation between testosterone and sleep quality, as assessed by the PSQI questionnaire. However, experiments have shown that total sleep deprivation leads to a decrease in testosterone levels in healthy men, whereas short-term sleep restriction does not always cause significant changes, emphasizing the importance of the first hours of sleep for hormone production. In addition, sleep disorders, such as apnea or fragmentation of nighttime rest, can lower testosterone levels. An optimal sleep architecture and adequate sleep duration are crucial for testosterone production and androgenic health. Although no studies have directly analyzed the relationship between testosterone levels and PSQI scores, establishing this relationship is important for understanding the impact of sleep quality on hormonal balance in men.

### 4.2. Cortisol

Our study showed a statistically significant negative correlation between body weight, height, and serum cortisol concentration in male participants. Based on an analysis of a sample of 1354 individuals, the researchers showed that height decreased with increasing blood cortisol concentration and that this relationship was continuous across the full range of concentrations assessed in both women and men. The authors of this study suggested that the HPA axis may mediate developmental mechanisms by limiting physical growth in favor of other competing processes [38].

The relationship between cortisol and obesity, often in the context of metabolic syndrome, has been studied in scientific literature. In our study, no significant correlations were found between cortisol levels and BMI, waist circumference, or adipose tissue, which may have been due to the small number of obese participants. A study by Garbellotto et al. also found no significant correlation between BMI and waist circumference and salivary cortisol concentration but found a significant reduction in cortisol concentration in men after weight loss [39]. Another study found a weak positive correlation between waist circumference and salivary cortisol concentration in men, and an increase in cortisol concentration with increasing BMI [40]. A 2016 meta-analysis did not confirm a relationship between BMI and morning cortisol levels, indicating the diversity of studies and the influence of other factors, such as sleep quality. However, high cortisol levels may exacerbate the complications of obesity [41]. In a study by Sofer et al., both serum and salivary cortisol concentrations were significantly lower in obese subjects than in those with a normal body weight. In addition, obese individuals show a weaker adrenal cortex response in the form of cortisol secretion in response to a low dose of ACTH [42]. In a study conducted among women by Schorr et al., the relationship between BMI and cortisol concentration was not linear but rather U-shaped on a graph. The highest serum cortisol concentrations were found at the lowest BMI values (approximately 15 kg/m^2^), and decreased up to a BMI of 30 kg/m^2^. Subsequently, as BMI increased, cortisol concentration increased but was significantly lower than that in the case of emaciation [43].

Our study did not show that the supply of macronutrients to the diet had a statistically significant effect on cortisol concentration. Martens et al. assessed the effect of the consumption of individual macronutrients on cortisol concentrations after a meal. Each participant first consumed an identical breakfast tailored to their individual energy requirements and then, under laboratory conditions, was given another meal consisting of pure protein, fat, or carbohydrates in the form of a shake with water and calorie-free flavoring. In the control group, the second meal consisted of flavored water only. The consumption of a protein or fat meal did not cause significant differences in cortisol concentrations compared with the control group. A significant difference was observed only after the consumption of a carbohydrate meal [44]. The intervention cited differed from our study, but the participants in our study had high protein and fat intake and low carbohydrate intake in their diet, which may suggest some consistency with the results of the above study.

A statistically significant relationship was observed between dietary cholesterol intake and blood cortisol concentration. A higher cholesterol intake is associated with a decrease in the concentration of this hormone. Cortisol is a steroid hormone; therefore, cholesterol is the substrate for its synthesis. In a study by Anderson et al., the effect of consuming meals rich or poor in cholesterol on changes in salivary cortisol concentrations was compared. An increase in the concentration of this hormone was observed after consuming a cholesterol-rich meal [45].

Our study showed that serum cortisol levels decreased with increased dietary folic acid intake. The relationship between B vitamins and cortisol levels is of particular interest because of their role in proper functioning of the nervous system and mood regulation. Administration of exogenous cortisol to subjects has been shown to result in a statistically significant reduction in serum folate and cobalamin concentrations [46]. This result aroused interest in the context of the inverse relationship that was investigated in a randomized study evaluating the effect of 16 weeks of vitamin B supplementation on salivary cortisol concentration. The preparation contained folic acid, vitamins B6, B12, C, D, and E, calcium, magnesium, potassium, and iron. The intervention did not show any changes in subjectively perceived stress levels, but cortisol concentrations upon waking up were higher in the group taking multivitamin preparations. Serum vitamin B6 and red blood cell folate concentrations were positively correlated with cortisol concentrations upon waking. Only the relationship between cortisol and folate levels was statistically significant, while the other two showed a clear trend [47]. These results did not correspond with the results of our study, in which a higher supply of folic acid, vitamins B6, B12, C, D, E, calcium, potassium, iron, and magnesium showed a negative trend in relation to serum cortisol concentration in male subjects.

Although our study showed a negative correlation between serum cortisol concentration and dietary vitamin A intake, no studies in the scientific literature have analyzed the significance of intake or supplementation of this vitamin or its precursor in the form of β-carotene on cortisol concentration in humans. An article examined the potential use of retinoic acid as a drug in the treatment of Cushing’s disease. This study showed that therapeutic doses (10–80 mg/day) of retinoic acid contributed to the normalization of hypercortisolemia in some patients [48].

Among the stimulants evaluated in our study, the consumption of caffeine in the form of energy drinks and supplements was statistically significant. More frequent use of these products was associated with an increase in the morning cortisol concentration in the blood plasma of men. A study by Gür et al. conducted on a group of footballers showed that caffeine intake has a significant impact on the body’s hormonal response and physical performance. Caffeinated coffee, decaffeinated coffee, pure caffeine in capsule form, and placebo capsules were considered. In the case of cortisol, the increase in cortisol concentration did not differ significantly between the coffee, decaffeinated coffee, and capsule groups, but was the lowest in the placebo capsule group. Caffeinated coffee results in greater exercise efficiency and higher testosterone concentrations [49]. Energy drinks may be of interest because of their characteristic combination of ingredients, such as caffeine and simple sugars, often also with taurine. A study by Sünram-Lea et al. assessed the effect of energy drinks on cortisol levels in firefighters [50]. Another study attempted to determine whether different combinations of caffeine (200 mg), taurine (2000 mg), and glucose (50 g), in doses similar to those found in popular energy drinks, would affect cortisol, among other things [51]. The cited studies did not show a significant effect of energy drinks on cortisol levels. Therefore, these reports do not correspond with our results. Another study emphasized that in addition to caffeine intake, tolerance developed among people who regularly use caffeine is also important. In such people, after caffeine was supplied to the body, a cortisol surge was observed, but it was significantly lower than that in people who had not used caffeine for five days [52]. Another study showed that with regular caffeine use, cortisol release was significantly higher in stressful situations [53].

This study also assessed the impact of alcohol consumption on cortisol levels. In a study by Badrick et al., salivary cortisol levels increased significantly in relation to weekly alcohol consumption in men. Furthermore, among people who abused alcohol, cortisol levels were observed to decrease more slowly than those who drank moderate amounts [54]. In our study, no statistically significant correlation was found between alcohol consumption and cortisol concentration; the correlation coefficient had positive values, and in the group that consumed alcohol most frequently, cortisol concentrations had the highest values, which may suggest a trend consistent with the aforementioned reports.

Our study also did not observe any statistically significant correlations or clear trends in smoking, marijuana use, or fasting blood cortisol levels. However, the literature indicates that the HPA axis may play an important role in nicotine addiction. In one study, in which a group of 20 men compared the effects of smoking cigarettes with high/low nicotine content, the researchers demonstrated that nicotine dose had a statistically significant effect on the hormonal response of the HPA axis. After smoking a cigarette with high nicotine content, the increase in ACTH concentration was significantly higher after just 12 min, and the increase in cortisol concentration increased significantly 20 min after smoking and reached its maximum value after 60 min. The authors pointed out that stimulation of the HPA axis by smoking may be an important mechanism associated with the development of addiction [55]. There are also scientific reports showing that smokers have chronically elevated cortisol levels (by approximately 35%) compared with non-smokers [56]. Another study showed the opposite effect associated with smoking cessation. It has been shown that 24 h abstinence causes a significant decrease in salivary cortisol levels and a reduction in HPA axis activity compared to a group that did not stop smoking [57]. Marijuana is often used to reduce stress; however, its effect on the HPA axis is unclear. There are reports that chronic use of cannabinoids results in a statistically significant reduction in cortisol levels and subjectively perceived stress compared to people who do not use this substance [58]. Another study found no significant changes in cortisol levels among marijuana users [59]. Despite the lack of statistical significance in the results obtained in our study, cortisol levels were relatively low in the group of most frequent marijuana users.

In the analyzed group, a negative correlation was observed between cortisol levels and the level of physical activity during leisure time. This may suggest that people who are more active during their free time have lower cortisol concentrations. This result is consistent with previous studies indicating that moderate physical activity may contribute to a reduction in perceived stress levels, which, in turn, leads to lower cortisol concentrations [60,61].

Spearman’s correlation analysis between cortisol concentration and the results of the Holmes and Rahe scale and PSS-10 did not show any significant relationships, which was also observed in other studies. In a study of women, higher PSS scores did not correlate with cortisol concentrations in hair, suggesting that subjective stress assessments are not always reflected in biochemical markers [62]. Similarly, the PRISME cohort study (n ≈ 4500) using PSS-4 found no association between stress and salivary cortisol concentrations, either in the morning or in the evening. The correlations were close to zero, and the mixed models did not confirm the influence of stress levels or changes in stress on cortisol secretion [63]. The authors emphasize that PSS should be treated primarily as an assessment of stress perception rather than as a direct indicator of HPA axis activation.

A weak negative correlation was found between cortisol concentration and sleep quality, suggesting that poor sleep is associated with lower hormone levels. The association was stronger in men under the age of 26 years, whereas no significant effects were observed in the older group. This result differs from previous studies indicating elevated cortisol concentrations in individuals with sleep disorders such as insomnia or depression [64,65]. The discrepancy may be due to the fact that healthy men without serious sleep disorders were studied and a single measurement of total serum cortisol was used, which did not capture the circadian rhythm and free fraction. The literature emphasizes the greater sensitivity of salivary cortisol measurements and CAR profile analysis. Chronic sleep disorders can lead to both weakening and an increase in the HPA axis activity. The relationship between sleep and the HPA axis is complex and depends on the nature and duration of the disorder. Furthermore, PSQI scores do not always correlate with endocrine markers; sometimes, no differences in CAR or diurnal cortisol profile are found between individuals with good and poor sleep quality [66].

The study measured total cortisol in the blood serum. It should be noted that only approximately 3–10% of circulating cortisol is free and biologically active, with the rest mainly bound to corticosteroid-binding globulin (CBG) and albumin. CBG concentration is affected by inflammation, medication, or estrogens, which can influence total cortisol levels independently of the HPA axis activity. Therefore, salivary cortisol measurements, which reflect free hormone concentrations, are more reliable for assessing the biological activity of glucocorticoids in many situations. In studies of sleep, stress, and circadian rhythm, measuring free cortisol, especially in a morning sample, is a more sensitive method and less susceptible to confounding factors [67,68,69]. However, in Poland, measurement of free cortisol in saliva is not yet routine.

This study has several limitations, including a relatively small sample size, which reduces the likelihood of detecting other significant correlations and may introduce potential subjectivity into the results. Nevertheless, the findings provide a valuable basis for designing and expanding future research and for selecting the appropriate research direction. Another limitation is the lack of consideration of food digestibility, which was not assessed due to the absence of this function in commonly available popular dietary software used to estimate nutrient intake in the male study group.

## 5. Conclusions

In conclusion, our study indicates that nutritional status and body composition play a key role in regulating testosterone and cortisol levels in young men. Higher protein, fat, and folic acid intakes were associated with higher testosterone concentrations, while increased body fat and caffeine consumption correlated with less favorable hormonal patterns. Physical activity had a beneficial effect on testosterone and cortisol balance; however, perceived stress and sleep quality showed no significant relationship. These findings underscore the importance of diet and lifestyle in maintaining hormonal homeostasis in healthy young males.

## Figures and Tables

**Table 1:** Correlations between dietary indices and selected nutrients in relation to free testosterone in the blood of male subjects (*n* = 40).

Parameter	Total	≤25 Years Old	≥26 Years Old
*n*	40	20	20
Index pHDI-10 *	0.093	0.040	0.152
Index nHDI-14 **	−0.05	−0.179	0.263
Energy [%] **	0.240	0.131	0.299
Protein[%] *	0.200	−0.022	0.485
Fat [%] **	0.241	0.115	0.366
SFA[%] **	0.189	0.065	0.328
MUFA[%] **	0.195	0.031	0.305
PUFA [%] **	0.122	0.216	0.068
Omega-6/omega-3 *	−0.019	0.121	−0.186
Cholesterol [%] **	0.023	−0.150	0.256
Carbohydrates [%] **	0.207	0.207	0.180
Fiber [%] **	0.200	0.332	0.048
Monosaccharides [%] **	0.184	0.133	0.214

* Pearson correlations. ** Spearman correlations. Correlation coefficients for *p* < 0.05 are marked in red.

**Table 2:** Correlations between the % consumption achievement of minerals in the diet and free testosterone in the blood of the male subjects (*n* = 40).

Mineral Component	Total	≤25 Years Old	≥26 Years Old
*n*	40	20	20
Na [%] *	0.315	0.317	0.311
K [%] **	0.097	0.006	0.220
Ca [%] *	0.295	0.151	0.430
P [%] *	0.214	0.089	0.364
Mg [%] **	0.271	0.306	0.311
Fe [%] *	0.303	0.091	0.484
Zn [%] *	0.186	0.120	0.274
Cu [%] **	0.272	0.307	0.257

* Pearson correlations. ** Spearman correlations. Correlation coefficients for *p* < 0.05 are marked in red.

**Table 3:** Correlations between the % consumption achievement vitamins in the diet and free testosterone in the blood of the male subjects (*n* = 40).

Vitamin	Total	≤25 Years Old	≥26 Years Old
*n*	40	20	20
B_1_ [%]	0.103	0.136	0.124
B_2_ [%]	0.040	−0.016	0.152
B_3_ [%]	0.046	−0.027	0.102
B_6_ [%]	−0.039	−0.186	0.094
Folic acid [%]	0.229	−0.002	0.518
B_12_ [%]	−0.042	−0.185	0.049
C [%]	0.085	0.170	0.010
A [%]	0.088	−0.086	0.310
D [%]	0.150	−0.025	0.318
E [%]	0.115	0.162	0.105

Spearman correlations. Correlation coefficients for *p* < 0.05 are marked in red.

**Table 4:** Correlations between diet indices and selected nutrients and cortisol in the blood of the male subjects (*n* = 40).

Parameter	Total	≤25 Years Old	≥26 Years Old
*n*	40	20	20
Index pHDI-10 *	−0.119	−0.121	−0.173
Index nHDI-14 **	0.027	−0.073	0.032
Energy [%] **	−0.124	−0.101	−0.140
Protein [%] *	−0.068	0.042	−0.229
Fat [%] **	−0.203	−0.271	−0.128
SFA [%] **	−0.301	−0.342	−0.306
MUFA [%] **	−0.151	−0.294	−0.090
PUFA [%] **	−0.177	−0.276	−0.134
Omega-6/omega-3 *	0.061	0.160	−0.200
Cholesterol [%] **	−0.282	−0.480	0.256
Carbohydrates [%] **	0.002	0.134	−0.122
Fiber [%] **	−0.019	−0.105	−0.021
Monosaccharides [%] **	−0.126	0.171	0.214

* Pearson correlations. ** Spearman correlations. Correlation coefficients for *p* < 0.05 are marked in red.

**Table 5:** Correlations between the % consumption achievement mineral in the diet and cortisol levels in the blood of male subjects (*n* = 40).

Mineral Component	Total	≤25 Years Old	≥26 Years Old
*n*	40	20	20
Na [%] *	−0.146	−0.162	−0.095
K [%] **	−0.087	−0.068	−0.232
Ca [%] *	−0.224	−0.246	−0.214
P [%] *	−0.187	−0.234	−0.242
Mg [%] **	−0.036	−0.068	−0.117
Fe [%] *	−0.240	−0.280	−0.263
Zn [%] *	−0.258	−0.353	−0.143
Cu [%] **	−0.138	−0.075	−0.263

* Pearson correlations. ** Spearman correlations.

**Table 6:** Correlations between the % consumption achievement vitamins in the diet and cortisol levels in the blood of the male subjects (*n* = 40).

Vitamin	Total	≤25 Years Old	≥26 Years Old
*n*	40	20	20
B_1_ [%]	0.010	−0.083	0.134
B_2_ [%]	−0.136	−0.441	0.033
B_3_ [%]	0.048	−0.150	0.167
B_6_ [%]	−0.003	−0.237	0.089
Folic acid [%]	−0.310	−0.489	−0.381
B_12_ [%]	−0.126	−0.376	0.156
C [%]	−0.097	−0.184	0.074
A [%]	−0.369	−0.646	−0.167
D [%]	−0.183	−0.403	−0.136
E [%]	−0.090	−0.166	−0.123

Spearman correlations. Correlation coefficients for *p* < 0.05 are marked in red.

**Table 7:** Correlations between free testosterone concentration and anthropometric measurements in relation to cortisol in the blood of male subjects (*n* = 40).

Cortisol [µg/dL]			
Parameter	Total	≤25 Years Old	≥26 Years Old
N	40	20	20
Free testosterone [pg/mL] *	−0.088	0.136	−0.342
Body weight [kg] *	−0.331	−0.529	−0.087
Body height [cm] *	−0.421	−0.534	−0.293
BMI [kg/m^2^] **	−0.073	−0.239	0.182
Waist circumference [cm] **	−0.117	−0.342	0.228
Body fat [%] *	0.001	−0.176	0.240
Body muscle tissue [%] *	−0.035	0.150	−0.265

* Pearson correlations. ** Spearman correlations. Correlation coefficients for *p* < 0.05 are marked in red.

**Table 8:** Correlations between cortisol concentration and anthropometric measurements in relation to free testosterone in the blood of the male subjects (*n* = 40).

Free Testosterone [pg/mL]			
Parameter	Total	≤25 Years Old	≥26 Years Old
*n*	40	20	20
Cortisol [µg/dL] *	−0.088	0.136	−0.342
Body weight [kg] *	−0.096	0.019	−0.040
Body height [cm] *	0.009	−0.252	0.198
BMI [kg/m^2^] **	−0.14	0.182	−0.269
Waist circumference [cm] **	0.007	−0.057	0.026
Body fat [%] *	−0.173	−0.070	−0.460
Body muscle tissue [%] *	0.176	−0.042	0.405

* Pearson correlations. ** Spearman correlations. Correlation coefficients for *p* < 0.05 are marked in red.

**Table 9:** The relationship between free testosterone, cortisol, and stress levels was assessed using the Holmes and Rahe scale and the Perceived Stress Scale (PSS-10) in the study group (*n*= 40).

Parameter	Holmes and Rahe	PSS-10
*n*	40	40
Free testosterone [pg/mL]	−0.10	−0.15
Cortisol [µg/dL]	−0.06	−0.04

Spearman correlations.

**Table 10:** Relationship between PSQI score and testosterone and cortisol concentrations in the study group, broken down by age (*n* = 40).

Parameter	Total	≤25 Years Old	≥26 Years Old
	40	20	20
Free testosterone [pg/mL]	0.09	−0.08	0.22
Cortisol [µg/dL]	−0.38	−0.45	−0.14

Spearman correlations. Correlation coefficients for *p* < 0.05 are marked in red.

**Table 11:** Relationship between activity indices and testosterone and cortisol concentrations in the study group, broken down by age (n = 40).

Parameter	Work Activity Index			Sports Activity Index			Leisure Activity Index		
	All	≤25 Years Old	≥26 Years Old	All	≤25 Years Old	≥26 Years Old	All	≤25 Years Old	≥26 Years Old
N	40	20	20	40	20	20	40	20	20
Free testosterone [pg/mL]	0.21	0.01	0.50	0.15	0.14	0.20	0.41	0.29	0.49
Cortisol [µg/dL]	0.04	0.13	−0.17	0.15	0.22	0.08	−0.17	−0.16	−0.35

Spearman correlations. Correlation coefficients for *p* < 0.05 are marked in red.

## Data Availability

The raw data supporting the conclusions of this article will be made available by the authors on request.

## Abbreviations

The following abbreviations are used in this manuscript:

SFA	saturated fatty acids
MUFA	monounsaturated fatty acids
PUFA	polyunsaturated fatty acids
